# A prospective comparative study of local infiltration versus adductor block versus combined use of the two techniques following knee arthroplasty

**DOI:** 10.1186/s42836-020-00034-8

**Published:** 2020-05-20

**Authors:** S. K. S. Marya, Deep Arora, Chandeep Singh, Shitij Kacker, Rahul Desai, Vikas Lodha

**Affiliations:** Bone & Joint Institute, Medanta, Medicity, H Baktawar Singh Road Sector 38, Gurugram, Haryana India

**Keywords:** Total knee arthroplasty, Adductor canal block, Local infiltration analgesia, WOMAC score

## Abstract

**Background:**

Pain management after total knee arthroplasty (TKA) is important as acute postoperative pain can affect patient’s ability to walk and participate in rehabilitation required for good functional outcome. This is achieved by effective intra-operative and post-operative analgesia to facilitate early recovery. Adductor canal block (ACB) and local infiltration analgesia (LIA) are analgesic regimens and commonly used for effective post-operative analgesia after TKA. Our aim was to compare the efficacy and outcomes of these two methods, combined and independently.

**Methods:**

Our study included 120 patients undergoing unilateral TKA, who were randomized into three groups: LIA (Group I), ACB (Group II) and combined LIA + ACB (Group III). Patients were operated by a single surgeon. The outcome was defined by post-operative analgesia achieved by the three techniques (measured by the NPRS) and amount of fentanyl consumed postoperatively. Secondary outcome was evaluated based on postoperative functional outcomes in terms of ability to stand, distance covered, range of motion of knee on the 1st post-operative day, complications and WOMAC (Western Ontario & McMaster Universities Osteoarthritis Index) scores.

**Results:**

All patients were available for analysis. Numerical Pain Rating Scale for pain showed significant differences at 24 h between Group I and Group II, with a *p* value of 0.018 (GroupI was better), significant differences were found at 24 h between Group III and Group II, with *p* values being 0.023 and 0.004 (GroupIII was better). No significant differences were found between Group I and Group III at 24 h. Total fentanyl consumption was significantly less in Group III than in Group I and Group II, with *p* value being 0.042 and 0.005, respectively (Group III was better and consumed less fentanyl). No significant differences were found in WOMAC scores between the three groups at baseline, 2 and 6 weeks after operation.

**Conclusion:**

In patients undergoing TKA, analgesic effect of combined ACB and LIA was superior, as indicated by reduced opioid consumption and no differences in functional outcomes and complications were observed as compared to separate use of the two techniques.

## Introduction

According to the World Health Organization (WHO), 9.6% of men and 18% of women older than 60 years of age worldwide have symptomatic OA, making this condition one of the most prevalent chronic conditions [1]. Total knee arthroplasty (TKA) is one of the most common elective orthopaedic procedures which has been proven and established to be a highly successful procedure in patients with severe knee osteoarthritis, being able to reduce pain and improve function and quality of life [2]. The number of knee replacement surgeries performed in the recent years have increased and are projected to increase 6-fold from 2005 to 2030 in the United States [3]^.^

There is an emphasis on postoperative analgesia and an ideal analgesia technique is required to provide adequate pain-free postoperative period, with knee mobility preserved, allow early return to activity, have lower rate of postoperative complications, lead to shorter hospital stay time and achieve better patient satisfaction [4, 5].

The various modalities of postoperative analgesia work by inhibiting pain receptors with different drugs acting in different modes. Patient-controlled analgesia (PCA), continuous epidural analgesia, peripheral nerve blocks, and local infiltration analgesia (LIA) are the usual pain management regimens [6, 7].

Patient-controlled analgesia (PCA) is a method that allows patients to administer the analgesic agent through an automated intravenous infusion pump [8, 9]. Although opioids provide effective analgesia, they are associated with side effects such as nausea, vomiting, pruritus, dizziness, urinary retention, sedation and constipation [10, 11]. The EA (epidural analgesia) is administered through a catheter placed in the epidural space, combination use of opioids and analgesics can be given but it is not without complications [12, 13]. The side effects, such as nausea, pruritus, hypotension, urinary retention, poor muscle control, delayed mobilization and the possibility of developing spinal hematoma in patients receiving LMWH (low molecular weight heparin) were higher (33: 100,000) [14], all of which have to be weighed against the benefits of EA (epidural analgesia).

Lumbar plexus block, also known as psoas compartment block or 3-in-1 block, comprises injection of a local anaesthetic on the fascial plane within the posterior aspect of the psoas major muscle [15]. This again leads to complete motor and sensory blockade, which causes quadriceps weakness and results in delayed mobilization and increased risk of falls. The femoral nerve block, most commonly used for postoperative analgesia, provides adequate pain relief but at the expense of motor blockade and weakening of the quadriceps [16]. The adductor canal block (ACB) or saphenous nerve block is a modified FNB, in which, under ultrasound guidance, a local anaesthetic is injected into the adductor canal deep to the sartorius muscle, resulting in an virtually purely sensory blockade. By targeting the adductor canal, the largest sensory branch of the femoral nerve, the saphenous nerve, the medial femoral cutaneous nerve, the articular branches of the obturator nerve, the vastusmedialis nerve, the medial articular nerve are blocked. Thus the motor function is largely spared and the technique allows for early ambulation and decreases the incidence of falls. Several studies have shown the advantages of ACB over FNB in terms of early mobilisation, quadriceps muscle strength preservation and better functional recovery [17, 18]. Local infiltration analgesia (LIA), or periarticular infiltration is another form of local analgesia given intra-operatively with a combination use of opiods, NSAIDS (non-steroidal anti-inflammatory drugs) and adrenaline. This technique has the benefits of simple administration, no need for motor blockade and less side effects. Several studies have demonstrated that both local infiltration analgesia (LIA) and adductor canal block (ACB) efficiently provide adequate analgesia after TKA, without causing quadriceps weakness and delayed mobilization. However, LIA is merely a single dose analgesia and a single-dose LIA may not be sufficient for postoperative analgesia after TKA [4]. ACB includes a single bolus dose and a continuous infusion through a pump and may be more effective in providing adequate analgesia without interfering with motor functions or early ambulation. Andersen et al [1] found that the combination of ACB and LIA offered better pain relief than LIA alone. Perlas et al [19] did not find any difference in pain relief between the combine use and LIA alone. Whether ACB plus LIA offers better analgesia and faster early postoperative recovery than LIA alone after TKA remains controversial. Thus, we conducted a prospective randomized study to compare the efficacy of the three modalities, that is, LIA versus ACB versus combination of ACB and LIA in terms of adequacy of postoperative analgesia and the functional outcomes after primary unilateral TKA.

## Materials and methods

This prospective study, upon approval by the institutional review board, was conducted in the authors’ affiliated institution from August 2017 to December 2018. Ethical clearance was obtained from the ethical committee and approval was obtained from the Institutional review board. Hundred and twenty subjects who were scheduled for elective unilateral TKA by a single orthopaedic surgeon were included in this study. Patients were randomly divided into three groups, with 40 subjects in each group. Group I (LIA group) Group II (ACB group) and Group III (LIA + ACB group). The patients were informed in detail about the study and written consent to participate in this study was obtained.

Eligibility criteria included (1) primary unilateral TKA, (2) age between 18 to 85 years, (3) no contraindications for either of the analgesic method, and (4) subjects were mentally sound and functionally rated grade I–III on the American Society of Anesthesiologists (ASA) scale. Exclusion criteria included (1) contraindications for peripheral nerve or neuraxial blockade, (2) history of allergy to drugs used in this study, (3) chronic pain requiring opioid medications, (4) psychiatric illness, and (5) patients receiving bilateral TKA and subjects refusing to be involved in the study.

After consent was obtained, participants’ baseline demographic information was collected and patients were randomly assigned to one of the three groups (Group I, II and III). All patients were operated under spinal anesthesia with 3 ml 0.5% heavy bupivacaine at L3–5 level. Surgery was performed using a femoral tourniquet. All surgeries were done through the same medial parapatellar approach to knee joint. All patients received posterior stabilized cemented implants. The LIA cocktail was prepared in operating room and was composed of 100 ml 0.2% Ropivacaine + Morphine 5 mg + 1 ml Adrenaline (1:1000) + Ketorolac 30 mg.

**Fig. 1:**
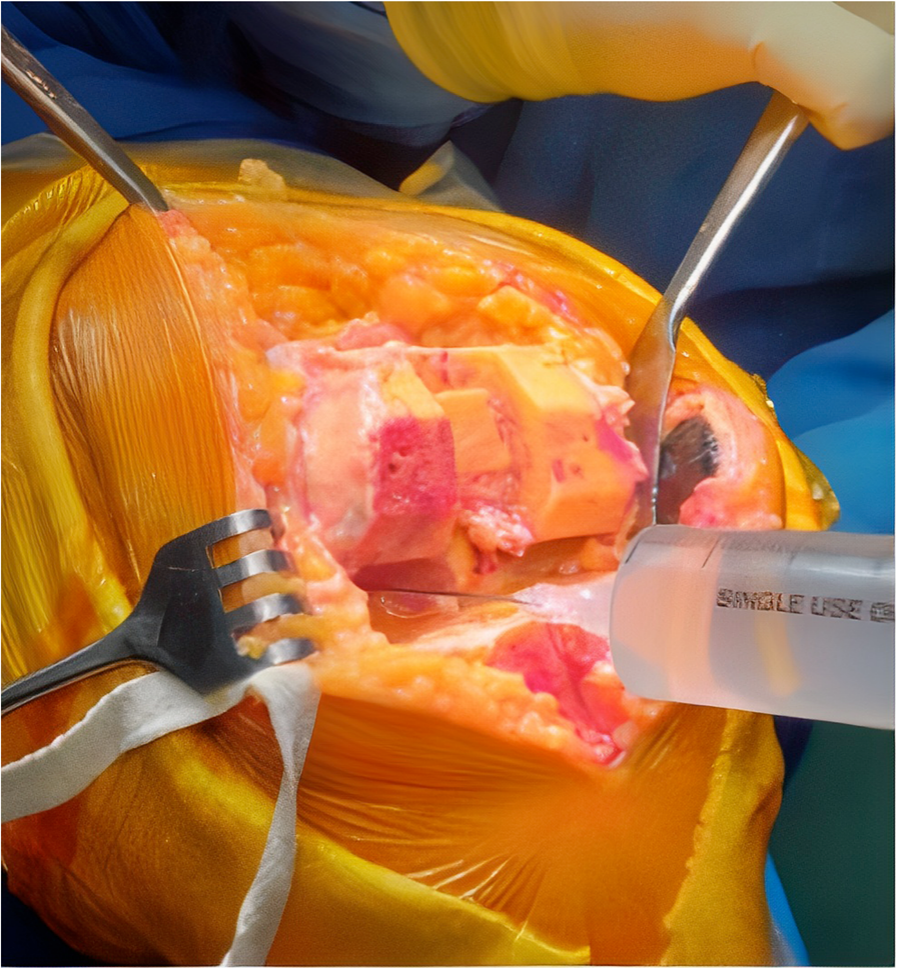
Intraoperative LIA (local infiltration analgesia)

The LIA was given in two stages. The prepared solution (50% of total volume) was first injected, after bone surface preparation and before component placement, into posteromedial capsule, attachment of residual posterior meniscal rim and posterior capsule, ACL femoral attachment and PCL tibial attachment, residual rim of the medial meniscus, posterolateral capsule, attachment of residual posterior rim of the lateral meniscus and posterior capsule, residual rim of lateral meniscus (Fig. 1). During the injection into the posterior areas of the knee, care was taken not to inject into popliteal artery. Aspiration was performed prior to any injection into the posterior region of the knee. Second injection of LIA cocktail (remaining 50% of total volume) was injected after component insertion but before wound closure while the cement was curing into the suprapatellar pouch, fat pad and retinacular tissues and subcutaneous tissues. Drain was used in all patients. At the end of surgery, the surgeon applied compression bandages covering the entire knee.

**Fig. 2:**
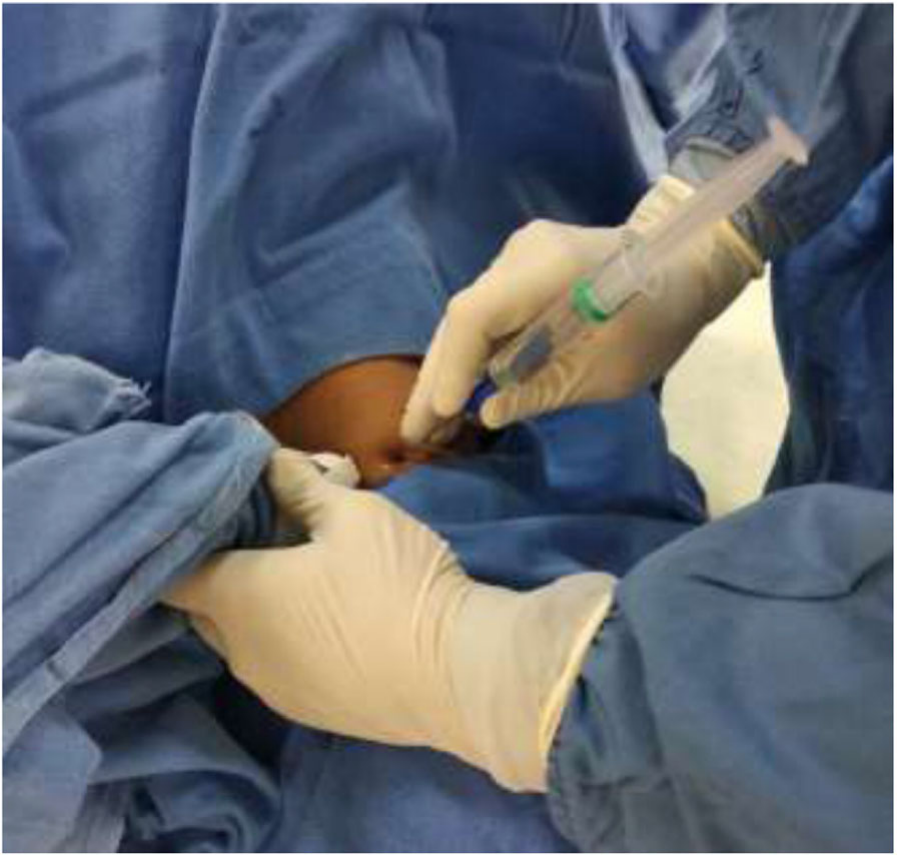
USG (Ultrasound-guided) ACB (Adductor canal block)

In the ACB group, ACB was usually administered after the surgery by the anesthesia team (Fig. 2). This block typically was performed with the patient in the supine position, with the thigh abducted and externally rotated to allow access to the medial thigh. It was performed using a high frequency ultrasound transducer. The goal was to place the needle tip just anterior to the femoral artery, deep to the sartorius muscle, and to deposit up to 20 ml of local anesthetic until its spread around the artery was confirmed with ultrasound visualization. The skin was disinfected and the transducer was placed antero-medially, approximately at the junction between the middle and the distal third of the thigh or somewhat lower. Once the pulsation of femoral artery had been identified, the probe was moved distally to trace the artery until it passed through the adductor hiatus to reach the popliteal artery. Then the probe was moved cephalad about 3–4 cm to obtain a good view in the axis of superficial femoral artery and the adjacent saphenous nerve. An adductor canal block was typically performed at this level. The needle was inserted in a lateral-to-medial orientation and advanced toward the femoral artery. Once the needle tip was visualized anterolaterally to the artery and after careful aspiration, 1–2 ml of local anesthetic was injected to confirm the right injection site which showed its spread around the femoral artery. Then 10–12 ml of Ropivacaine was injected at a rate of 5 ml/hr.

In the third group (LIA + ACB group), a single shot of 50 ml LIA was given intraoperatively after bone surfaces had been prepared into the posteromedial capsule, attachment of residual posterior meniscal rim and posterior capsule, residual rim of the medial meniscus, posterolateral capsule, attachment of residual posterior rim of the lateral meniscus and posterior capsule, residual rim of lateral meniscus and before components were inserted. Postoperatively ACB was given as in group II.

There was not substantial difference in the operating time among the three methods, which lasted for about 45 to 55 mins. LIA had the shortest time, taking approximately 50 mins as 50 ml of LIA was injected after preparation of bone surfaces and the injection of the rest 50 ml after the final implantation. With the group II (ACB), when the surgeons operating time was lesser than the group I, the operating room time was longer as the procedure of ACB took roughly 10–15 min for our anesthesia team. The group III had the longest operating room as the surgeon had to deliver 50 ml of LIA after preparation of bone surfaces and the anesthesia team had to perform ACB.

The postoperative pain control regime consisted of a PCA (patient controlled analgesia). In the intensive care unit, all patients were intravenously administered PCA fentanyl. At the outset, a bolus of 3 ml (30 mcg) was given. It was later given at a lockout interval of 20 mins and administered over a period of 48 h postoperatively.

The primary outcome of the analgesic effectiveness was measured in terms of Numerical Pain Rating Scale (NPRS) scores by assessing and asking the patients in the first 24 h of surgery at fixed intervals of 6, 12 and 18 h. The total amount of fentanyl consumed as rescue analgesia to support primary block was recorded at the start, 6, 12 and 24 h. Numerical Pain Rating Scale score (NPRS) is a measure of pain intensity, which is a whole number (0–10 integers) a respondent selects. The pain was rated on a 0-to-10 scale, with 0 representing no pain and 10 indicating extreme pain. It is effective, reliable and takes only few minutes to complete.

On the first post-operative day, ambulation ability and mobilization achieved by patients were assessed in terms of ability to stand, distance covered and range of knee flexion measured by goniometer. Adverse effects resulting from analgesics in the form of nausea, vomiting episodes, pruritus, quadriceps weakness, any unwanted cardiovascular and neurological events, DVT (deep vein thrombosis) and falls were noted. The patients were discharged on the 4th post-operative day, the criteria for discharge included that (1) the patients were ambulatory and (2) were able to manage their day-to-day activities with minimal pain. The primary outcome was based on the postoperative pain control and the amount of fentanyl consumed. Further functional assessment of patients was done 2 and 6 weeks after operation in terms of WOMAC (Western Ontario & McMaster Universities Osteoarthritis Index) score.

The analysis included profiling of patients on different demographic data. Quantitative data were presented as means and standard deviation. Qualitative/categorical data were expressed as absolute numbers and proportions. Cross tables were generated and chi square test was used for testing of significance. Student *t* test was used for comparison of quantitative parameters of outcomes. A *P* value < 0.05 was considered statistically significant. SPSS software package (Version 24.0) was used for statistical analysis.

## Results

**Fig. 3:**
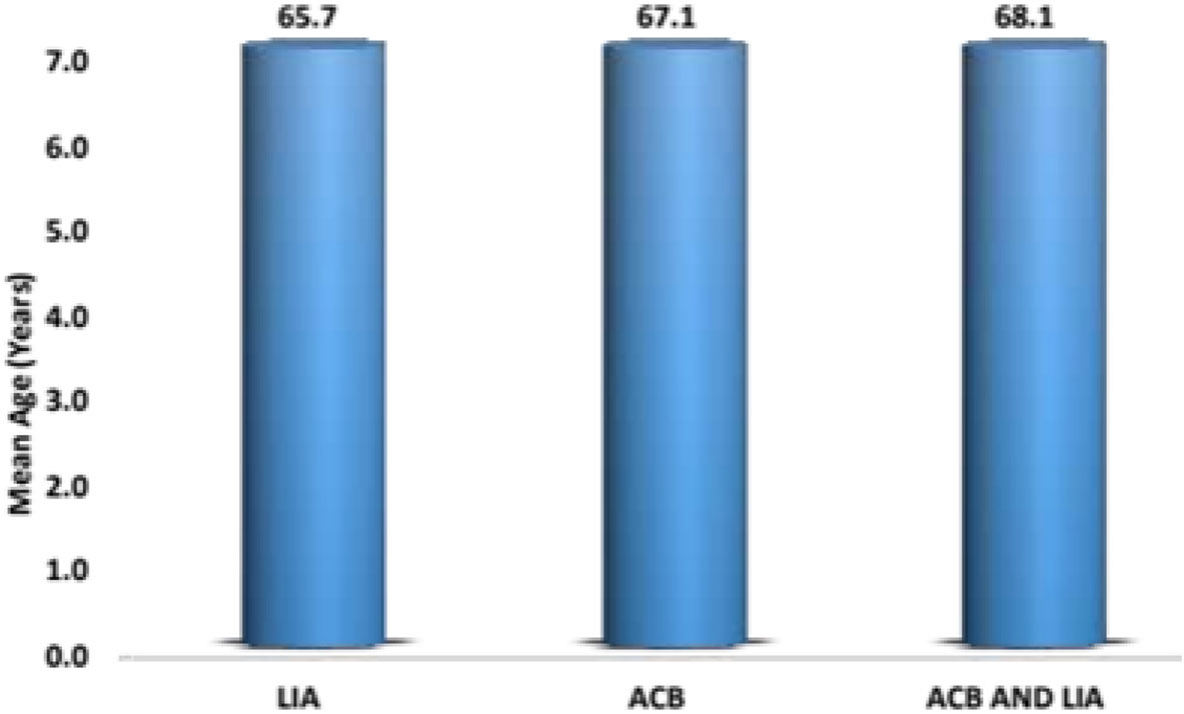
Diagram showing mean age in all groups

The differences between the three groups in demographic data (including age, gender) were not statistically significant (Fig. 3 and Table 1). The ASA (all patients were of grade I, II or III), site of surgery (all receivedunilateral total knee replacement). There was no substantial difference in operating time among the three techniques, with the differences ranging fromjust 6–8 min on the either side of the SD curve. This time difference did not exert any effect on the patients’ functional outcomes. 11111

**Table 1:** Comparison of study parameters between groups (Pair-wise)

	**LIA** **(Group I) (Mean ± SD)**	**ACB** **(Group II) (Mean ± SD)**	**ACB AND LIA** **(Group III) (Mean ± SD)**	***p-*value**		
				**Group** **(I vs. II)**	**Group** **(I vs. III)**	**Group** **(II vs. III)**
**Age (Years)**	65.7 ± 10	67.2 ± 8	68.2 ± 8.3	0.743	0.431	0.868
**Flexion of knee on 1st POD**	78.3 ± 6.4	77 ± 7.6	80.3 ± 4.2	0.641	0.324	0.054
**Pain Score**						
**0 HR**	2 ± 0.7	2.3 ± 1.5	1.8 ± 1	0.523	0.709	0.149
**6 HR**	2.8 ± 1.2	2.8 ± 1.2	2.3 ± 1.3	1.000	0.166	0.166
**12 HR**	2.5 ± 1.2	3 ± 1.1	2.4 ± 1	0.078	0.873	0.023*
**24 HR**	2.7 ± 1.1	3.4 ± 1	2.6 ± 1.2	0.018*	0.866	0.004*
**Change (0 to 24 h)**	0.70 ± .12	1.1 ± 1.69	0.77 ± 1.44			
***p*–value of Change**	0.001*	0.0001*	0.002*			
**Consumption of Fentanyl**						
**0–6 HR**	6 ± 4.1	7.4 ± 5.6	3.5 ± 3.8	0.360	0.042*	0.001*
**6–12 HR**	8.1 ± 6.1	10.6 ± 7.1	5.7 ± 4.8	0.172	0.179	0.001*
**12–24 HR**	9.2 ± 6.7	11 ± 6.3	6.6 ± 5.5	0.367	0.160	0.005*
**Change (0–6 to 12–24 h)**	3.20 ± 5.99	3.67 ± 5.82	3.15 ± 5.17			
***p*–value of Change**	0.002*	0.0001*	0.0001*			
**WOMAC Score**						
**Pre-operative**	61.7 ± 6.6	62.9 ± 8.1	61.5 ± 10.1	0.799	0.998	0.761
**2 Weeks follow up**	55.2 ± 6.4	55.9 ± 7.8	55 ± 9.5	0.919	0.989	0.856
**6 weeks follow up**	27.7 ± 5.2	27.3 ± 5.8	29 ± 6.6	0.950	0.574	0.392
**Change (Pre Op to 6 weeks)**	33.97 ± 4.45	35.57 ± 4.81	32.52 ± 5.13			
***p*–value of Change**	0.0001*	0.0001*	0.0001*			

*p-value < 0.005, statistically significant; #-Independent *t*-test; ##-One WAY ANOVA Tests

**Fig. 4:**
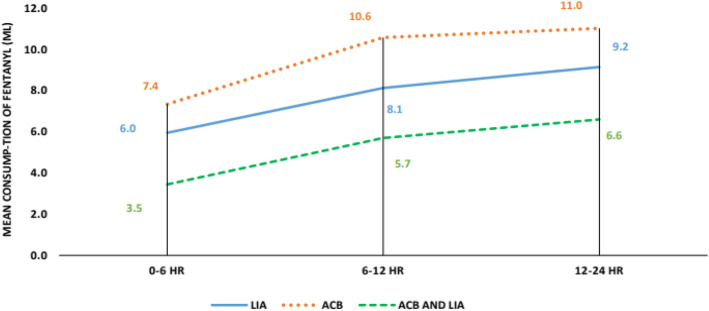
Mean consumption of fentanyl [in millilitres (ml)]

**Fig. 5:**
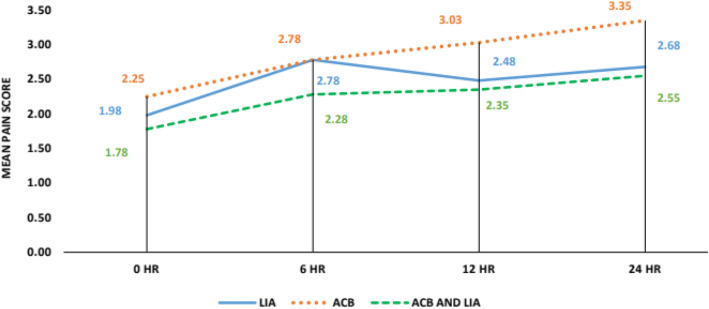
Mean pain score

**Fig. 6:**
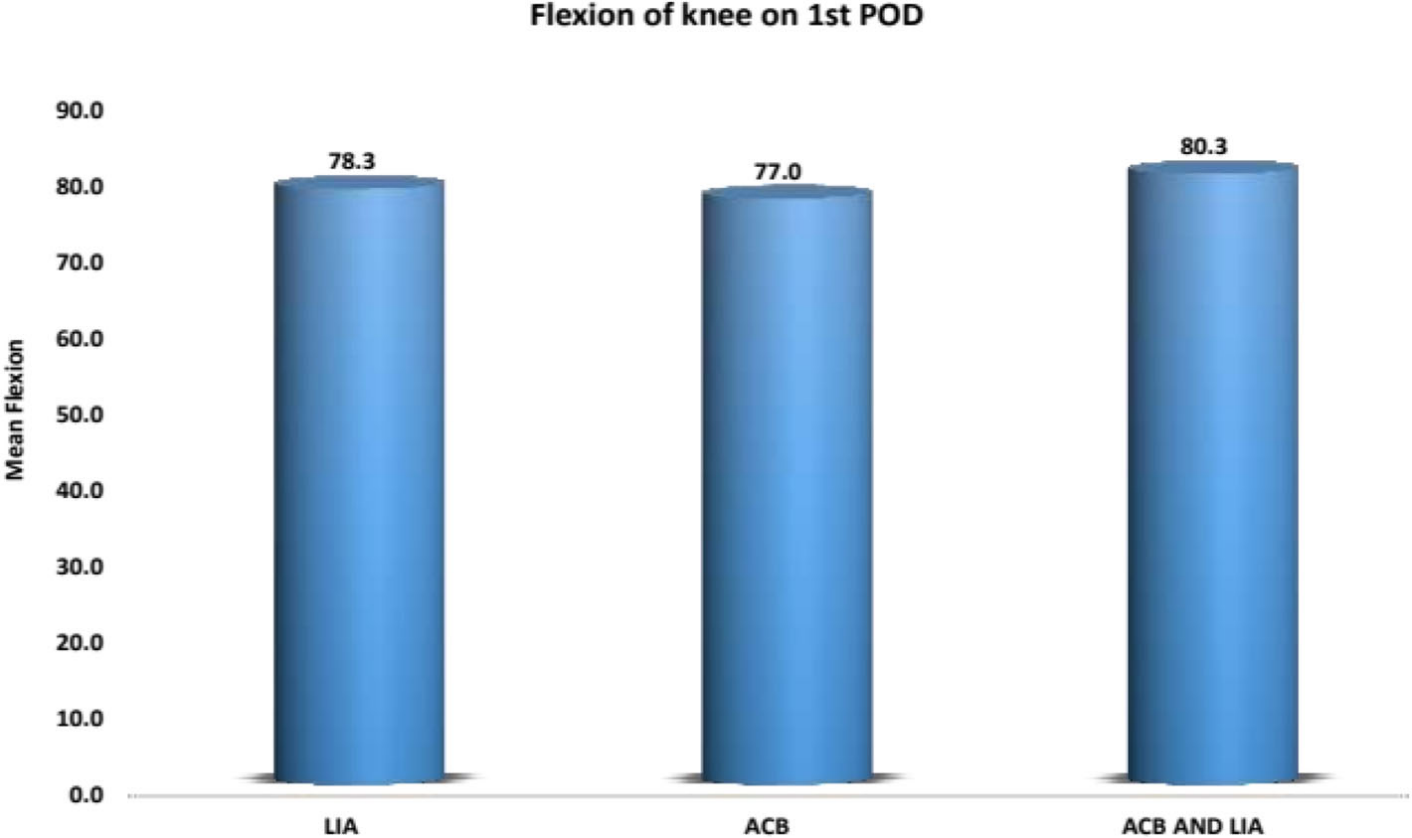
Flexion of knee on the 1st POD (post-operative day)

**Fig. 7:**
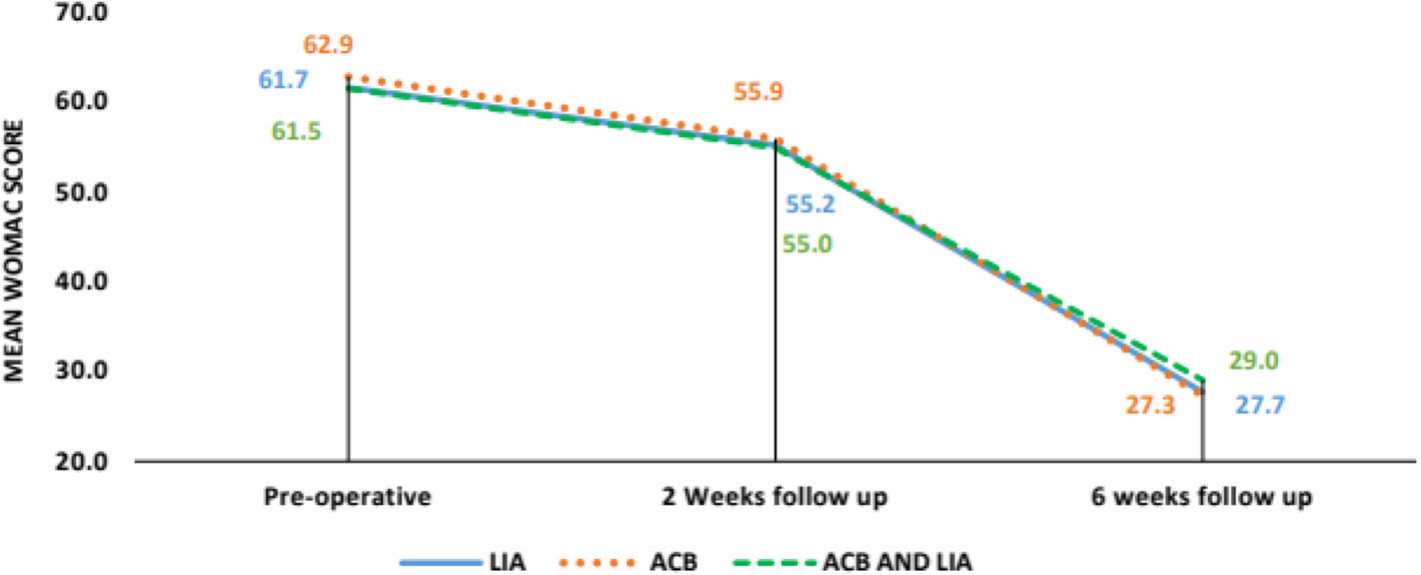
Mean WOMAC (Western Ontario & McMaster Universities Osteoarthritis Index) scores

**Fig. 8:**
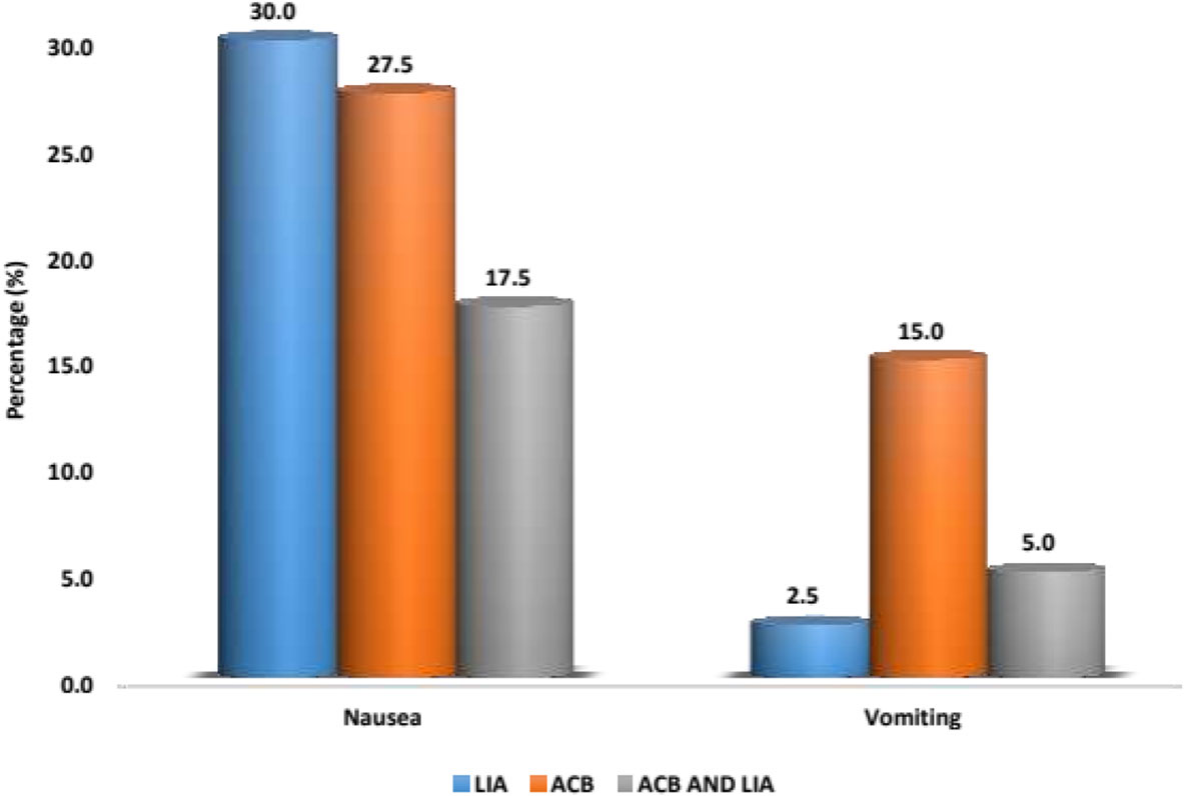
Percentages of nausea and vomiting in each group

The length of hospital stay (5 to 6 days) was almost the same with all patients. Post-operative fentanyl consumption was taken at 0–6 h, 6–12 h, and 12–24 h. Significant differences were noted at 0–6 h between LIA and combination group, with *p* value being 0.042 (combination group was better) and significant differences were found at 0–6 h, 6–12 h and 12–24 h between combination group and ACB group with *p*-values being 0.001, 0.001 and 0.005 respectively (Combined group consumed less fentanyl). No significant differences were found between LIA and ACB group till 24 h as shown in Fig. 4. Post-operative pain was rated *as per* NPRS scoring by patients themselves. Significant differences were noted at 24 h between the LIA and ACB group with *p* value being 0.018 (LIA worked better) and significant differences were found at 12 h and 24 h between the combination group and ACB group, with *p* values being 0.023 and 0.004 respectively (Combined group was better). No significant differences were found between the LIA and combined group till 24 h as in Fig. 5. Mean value of knee range of motion and distance covered at the 1st POD were found to be 78.25 degrees and 5.9 m for LIA group, 77 and 5.8 for ACB group, 80.25 and 6.15 for combination group, with no significant differences found between any groups (Fig. 6). Patients in group I had a mean WOMAC score of 61.65 before operation, 55.23 and 27.68 at the 2nd and 6th week after operation; group II had a mean WOMAC score of 62.85 before operation, 55.93 and 27.28 at the 2n and 6th weeks after operation; group III had a mean WOMAC score of 61.53 before operation and 54.98 and 29.00 at the 2nd and 6th week after operation. No significant differences were found between the groups at baseline or 2 and 6 weeks after opration in terms of the total WOMAC score. The comparison of all the above parameters pair-wise and through One way ANOVA test is depicted in Tables 1 and 2. All groups improved from baseline till the 2nd and then 6th week after operation (Fig. 7). We did not find any significant differences in incidence of nausea or vomiting and pruritus during postoperative period. (Fig. 8). No falls were recorded in either group. No major complications, either systemic or local, related to the anesthetic use were seen. The comparison of the complications is shown in Table 3. In our institution, we give a package to our arthroplasty patients, which includes everything from admission to discharge. It would be difficult for us to break up and analyze the cost for each method used in our study. However this needs further evaluation.

**Table 2:** Comparison of mean value of parameters between three groups (One Way ANOVA Test)

	**LIA** **(Group I)** **(Mean ± SD)**	**ACB** **(Group II)** **(Mean ± SD)**	**ACB AND LIA** **(Group III)** **(Mean ± SD)**	***p*-value of between group comparison based on ANOVA**
**Age (Years)**	65.7 ± 10	67.2 ± 8	68.2 ± 8.3	0.460
**Male, n (%)**	15 (37.5%)	10 (25.0%)	14 (35.0%)	0.450
**Flexion of knee on 1st POD**	78.3 ± 6.4	77 ± 7.6	80.3 ± 4.2	0.066
**Pain Score**				
**0 HR**	2 ± 0.7	2.3 ± 1.5	1.8 ± 1	0.173
**6 HR**	2.8 ± 1.2	2.8 ± 1.2	2.3 ± 1.3	0.114
**12 HR**	2.5 ± 1.2	3 ± 1.1	2.4 ± 1	0.019*
**24 HR**	2.7 ± 1.1	3.4 ± 1	2.6 ± 1.2	0.003*
**Consumption of Fentanyl**				
**0–6 HR**	6 ± 4.1	7.4 ± 5.6	3.5 ± 3.8	0.001*
**6–12 HR**	8.1 ± 6.1	10.6 ± 7.1	5.7 ± 4.8	0.002*
**12–24 HR**	9.2 ± 6.7	11 ± 6.3	6.6 ± 5.5	0.007*
**WOMAC Score**				
**Pre-operative**	61.7 ± 6.6	62.9 ± 8.1	61.5 ± 10.1	0.739
**2 Weeks follow up**	55.2 ± 6.4	55.9 ± 7.8	55 ± 9.5	0.859
**6 weeks follow up**	27.7 ± 5.2	27.3 ± 5.8	29 ± 6.6	0.393

*p-value < 0.005 is statistically significant

**Table 3:** Adverse effects

	**LIA** **(Group I) n (%)**	**ACB** **(Group II) n (%)**	**ACB AND LIA (Group III)** **n (%)**	***p-*value**			
				**Group** **(I vs. 2)**	**Group** **(I vs. III)**	**Group** **(II vs. III)**	**ANOVA**
**Any Adverse effects**	**13 (32.5)**	**17 (42.5)**	**9 (22.5)**	0.314	0.319	0.047	0.161
**Nausea**	12 (30.0)	11 (27.5)	7 (17.5)	0.806	0.192	0.287	0.393
**Vomiting**	1 (2.5)	6 (15.0)	2 (5.0)	0.049	0.559	0.138	0.080
**Pruritus**	None	None	None	–	–	–	–
**Fall**	None	None	None	–	–	–	–
**Distal neuro- vascular deficit**	None	None	None	–	–	–	–
**DVT**	None	None	None	–	–	–	–
**Convulsion**	None	None	None	–	–	–	–
**Cardiovascular Complications**	None	None	None	–	–	–	–

## Discussion

This was a prospective, randomized, multimodal study in which we compared the analgesic effects of three modalities, i.e., LIA, ACB and combined use of LIA and ACB. We found that all the three techniques could achieve good postoperative pain control, good functional outcome and patient satisfaction albeit the combined group patients outperformed the other two groups. The group I (LIA) was better than the group II (ACB) patients. In patients undergoing TKA, all the three techniques can be utilized for attaining pain relief when combined with multimodal analgesic regime. There was a significant difference in fentanyl consumption and pain score between the groups and group III did better than the other two groups. The primary endpoint, total fentanyl consumption was lower and pain control was better in Group III (ACB + LIA) than in Group I (LIA) and Group II (ACB) at 24 and 48 h, post-operatively. LIA was able to provide greater pain relief and led to less fentanyl consumption than ACB at 12 and 24 h, postoperatively. No significant differences were found in opioid consumption in some another studies, such as a study by Lykke Andersen et al [1] (2012) who compared LIA + ACB and ACB alone and LIA alone. A study by Qiujuan et al [9] (2017) showed opioid consumption was significantly more in LIA group than in ACB + LIA group. The range of motion of the knee joint, the postoperative WOMAC scores and the adverse effects were similar among the three groups (LIA + ACB, ACB alone and LIA alone). These results may have significant implication since better pain relief and greater range of motion of the knee joint can enhance early mobilization and facilitate ambulation after the surgery. The studies by Chavis [6] and Hawker [7] indicated that poor control of pain or persistence of pain during the hospital stay may result in failure to achieve the desired functional outcomes and may contribute to increased incidence of postoperative complications such as pneumonia, deep vein thrombosis (DVT) or pulmonary embolus (PE) [6, 7]. Thus adequate postoperative pain control is crucial during early postoperative period. Effective early mobilization of the patients in the first 24 h after TKA has been shown to help increase range of motion, enhance muscle strength and gait control and reduce length of hospital stay [20]. Sawhney et al [2], in a prospective RCT study, compared the ACB plus LIA and LIA alone and suggested that there was no substantial difference between the two groups in terms of distance walked. LIA has come to be accepted and is being widely used because of its simplicity and effectiveness in providing postoperative analgesia and promoting early mobilization without affecting the quadriceps muscle strength [15–17, 21]. The drawback of LIA is that it is a single shot analgesia that is administered intra-operatively and lasts only for the first 6 to 12 h after TKA [18, 21]. The adductor canal is an aponeurotic space in the thigh, extending from the apex of the femoral triangle to the adductor hiatus [14]. Most nerves in the adductor canal are sensory nerves to the knee joint [13]. The ACB, therefore, seems to induce sensory anesthesia to the knee with potentially limited impact on motor function. Andersen et al [1] suggested that the ACB was effective as a rescue block when LIA failed to control pain. Perlas et al [19] demonstrated that the addition of ACB to LIA was associated with further improvement in early ambulation benchmarks and a higher rate of home discharge compared with LIA alone. Besides, Andersen et al [1] conducted an RCT including 40 patients undergoing TKA (20 patients receiving ACB in addition to LIA and 20 patients receiving LIA) and found that all patients in the ACB + LIA group were able to ambulate on the day of surgery against 13 patients in the LIA group (*p* = 0.004). It appears that the addition of a selective ACB to LIA is preferable to LIA alone for early postoperative rehabilitation. Several studies demonstrated that the analgesic effect of LIA lasted for about 6–12 h postoperatively [8]. The studies regarding ACB have shown that the duration of sensory blockade was 18–22 h [21]. These data were consistent with the present study in which postoperative pain score and fentanyl consumption in group III were found to be less than in group I and II. There was no variability in the skill of performing ACB as it was administered by the same anesthetic team. The combination of ACB and LIA has the advantages of both techniques with an already-established multimodal regimen that includes opioids and anti-inflammatory drugs. The preservation of quadriceps strength without the risk of perioperative leg weakness could potentially reduce the risk of falls. Our results were similar to a study by Sawhney et al [2], which demonstrated local infiltration analgesia could achieve greater pain relief at rest and movement compared with ACB. Postoperative quadriceps strength in Group III was similar to the other two groups, and this may be ascribed to the low levels of postoperative pain, which were seen in all three groups. The motor preservation also suggested that ACB did not interfere with quadriceps strength, as noted in previous studies.

This study has several limitations: (1) the sample size was too small to detect significant differences in the complications; (2) Post-TKA function exercise is a long-term process, and blocking effect diminishes over time (3) The study of pain management was conducted 24 to 48 h after surgery and (4) LIA given intra-operatively covered all the quadrants while ACB involves only the antero-medial aspect of the knee.

## Conclusion

In conclusion, the analgesic effect of combination use of LIA + ACB in TKA was better and was associated with a greater reduction of opioid consumption than LIA alone and ACB alone without any significant difference in complications. A single-injection LIA plus ACB using multimodal analgesia for TKA was associated with a greater reduction of fentanyl consumption than ACB alone or single injection of LIA. Furthermore, it provided superior analgesia and mobilization in the first 18 h after operation.

## Data Availability

Data were sufficient to support the study and more studies are needed in this area for final conclusion. The data are available through PubMed.

## Abbreviations

ACB: Adductor canal block
DVT: Deep vein thrombosis
LIA: Local infiltration analgesia
NPRS: Numerical Pain Rating Scale score
OA: Osteoarthritis
ASA: American Society of Anesthesiologists
PCA: Patient controlled analgesia
PE: Pulmonary embolism
POD: Post-operative day
TKA: Total knee arthroplasty
WOMAC: Western Ontario & McMaster Universities Osteoarthritis Index
